# Asymptomatic schwannoma of the heart

**DOI:** 10.1186/1749-8090-2-1

**Published:** 2007-01-04

**Authors:** Sarah A Early, Jonathan McGuinness, John Galvin, Maria Kennedy, John Hurley

**Affiliations:** 1 Department of Cardiothoracic Surgery, Mater Misericordiae University Teaching Hospital, Ireland; 2 Department of Cardiology, Mater Misericordiae University Hospital Teaching, Ireland; 3 Department of Pathology, Mater Misericordiae University Teaching Hospital, Ireland

## Abstract

We present a case of an asymptomatic right atrial mass detected on a screening ECHO. Pre-operative imaging and intraoperative frozen section suggested an atrial myxoma, but the extracardiac nature of the mass and its adherence to the right superior pulmonary vein and interatrial septum were inconsistent with this. Detailed histological assessment confirmed the diagnosis of atrial schwannoma. Limited case reports have shown complete resection is curative.

## Background

A 57 year old male was admitted with haematemesis. He had a previous history of peptic ulcer disease and had a Billroth's Type II procedure performed 10 years ago. As part of his work-up, a transthoracic ECHO was performed and a mass in the right atrium was identified. The patient denied any cardiovascular symptoms and his medical history was otherwise unremarkable.

**Figure 1:**
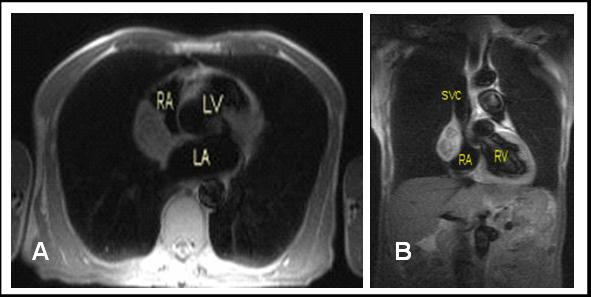
MRI heart. A. Sagittal. B. Coronal. RA = Right atrium, LA = Left atrium, LV = Left ventricle, SVC = Superior vena cava. The close proximity of the tumor to the right atrium and extension towards a thickened interatrial septum can be seen.

The patient was tall and thin with no murmurs and apart from normochromic normocytic anaemia, routine blood tests, ECG, and chest x-ray were normal. He had a gastroscopy which revealed gastritis that settled with intravenous omeprazole. A cardiac MRI depicted a 4.3 × 5.2 cm mass in the posterolateral wall of the right atrium extending to the interatrial septum which was thickened. There was 50% compression of the superior vena cava. The mass showed heterogenous very mild enhancement (Figure 1A). An angiogram demonstrated normal left ventricular function and coronary arteries, with a tumour blush in the right atrium. Two biopsies were inconclusive, and an atrial myxoma was suspected.

**Figure 2:**
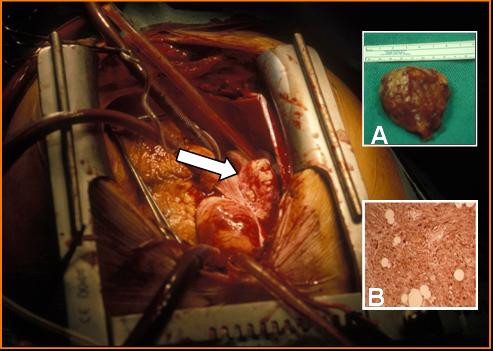
Intra-operative picture depicting extra-cardiac tumor (arrow). Inset A, Resected specimen. Inset B, Immunohistochemistry showing strong diffuse positivity for S-100.

At surgery, a solid rubbery 8 cm × 8 cm mass was found outside the heart within the pericardium on the lateral wall of the right atrium, and extending to the right superior pulmonary vein (Figure 2). With dissection a clear plane was noted between the right atrial wall, the superior right pulmonary vein, and the tumor, but the mass was tightly adherent to the interatrial septum. Using bicaval cannulation the patient was placed on cardiopulmonary bypass and the right atrium was opened but despite the echo and angiogram findings, no intra-atrial tumor was seen. The interatrial septum was opened to assess the left atrium which was also clear of tumor, and biopsies of the thickened atrial septum were taken prior to primary closure. The mass was resected en-bloc externally, close to the interatrial septum (Figure 2A). Frozen section was performed which suggested an atrial myxoma. The patient made an uncomplicated post-operative recovery.

Subsequent microscopic sectioning of the mass revealed an extensively infiltrating, focally cellular spindle cell tumour with a low mitotic index (ki 67 and immunostaining). A definitive diagnosis of an atrial schwannoma was made due to the strong diffuse uniform staining for s100beta typical for atrial schwannoma and the characteristic immunohistochemical appearance. (Figure 2B). The differential diagnosis included paraganglioma, neurofibroma and myxoma. It was not a paraganglioma as no well-formed ganglion cells were present. The diagnosis of neurofibroma was excluded due the presence of numerous blood vessels within the tumour. The weak CD 34 staining and S100beta staining excluded an atrial myxoma. The resection margins were clear and interatrial septal biopsies were normal.

## Discussion

Primary neurogenic neoplasms of the heart are very rare. The majority of these schwannomas or neurilemomas, as they are also called, occur on the right side of the heart and appear in close proximity to the interatrial septum. The reason postulated for this is that the origins of cardiac schwannomas are from cardiac branches of the vagus nerve and cardiac plexus [1,2]. Diagnosis can only be made by histological examination, frozen section is not adequate as was demonstrated in our case. Positive immunohistochemical analysis for S-100 protein supports the Schwann cell origin of the tumour [3].

A review the literature revealed that there have only been 14 previous cases of atrial schwannomas reported. Of these 71 % (10) of schwannomas have been in females and 9 were found attached to the right atrium in the position seen here. Two cases were discovered at autopsy while 3 were found after abnormal cardiac silhouettes were picked up on chest x-ray. The ages of the patients ranged from 12 to 72 years. Cardiac MRI and angiogram can help to define the morphology and extent of these lesions. The majority required cardiopulmonary bypass for assessment and complete resection of the tumour. The prognosis is excellent for those with complete tumour clearance and the risk of local invasion warrants surgical excision and reconstruction as required [4].

Our case was unusual in that it was only discovered after the patient had a screening ECHO. He was clinically asymptomatic. Cardiac MRI is helpful in diagnosing these tumours, specifically if the tumour is closely associated with the right atrium and interatrial septum[5]. However, full histological examination is required to confirm the diagnosis. Complete surgical excision is the procedure of choice, with reconstruction as required. Prognosis is excellent with complete excision.
